# Mechanical Properties and Microstructure of Geopolymer-Based PFSS Synthesized from Excavated Loess

**DOI:** 10.3390/ma18010030

**Published:** 2024-12-25

**Authors:** Shujie Chen, Hengchun Zhang, Zhengzhou Yang, Chao Feng, Yao Wang, Demei Yu, Tengfei Fu, Feng Zhang, Xia Huang

**Affiliations:** 1 College of Transportation and Civil Engineering, Fujian Agriculture and Forestry University, Fuzhou 350108, China; 5221342021@fafu.edu.cn (S.C.); 5221342046@fafu.edu.cn (Z.Y.); futengfei@fafu.edu.cn (T.F.); 2 CSCEC Strait Construction and Development Co., Ltd., Fuzhou 350015, China; 0591502@sina.com (Y.W.); zimu328@126.com (F.Z.); 13215985453@163.com (X.H.); 3 China State Construction Engineering Corporation, Beijing 100029, China

**Keywords:** pre-mixed fluidized solidified soil, water–solid ratio, curing agent dosage, workability and mechanical strength, microstructure

## Abstract

Pre-mixed fluidized solidified soil (PFSS) has the advantages of pumpability, convenient construction, and a short setting time. This paper took the excavated loess in Fuzhou as the research object and used cement–fly–ash–ground granulated blast furnace slag–carbide slag as a composite geopolymer system (CFGC) to synthesize PFSS. This study investigated the fluidity and mechanical strength of PFSS under different water–solid ratios and curing agent dosages; finally, the microstructure of the composite geopolymer system–pre-mixed fluidized solidified soil (CFGC-PFSS) was characterized. The results showed that when the water–solid ratio of PFSS increased from 0.46 to 0.54, the fluidity increased by 77 mm, and the flexural strength and compressive strength at 28 d decreased to 450.8 kPa and 1236.5 kPa. When the curing agent dosage increased from 15% to 25%, the fluidity increased by 18.0 mm, and the flexural strength and compressive strength at 28 d increased by 1.7 times and 1.6 times. A large number of needle-like AFt, C-S-H gel, and C-(A)-S-H gel coagulate with soil particles to form a three-dimensional reticular structure, which is the mechanism of the strength formation of PFSS under the action of CFGC.

## 1. Introduction

The United Nations Environment Programme reported a significant rise in industrial waste due to the rapid growth of the global construction industry. This included substantial amounts of materials such as fly ash (FA), ground granulated blast furnace slag (GGBFS), calcium carbide slag (CS), and various types of construction debris [1,2,3]. The generation of these solid wastes was projected to reach 3.8 billion tons by 2050 [4].

Currently, supplementary cementitious materials like GGBFS and FA have been widely researched and applied in the production of concrete and soil solidification [5,6,7,8,9,10]. CS, a byproduct mainly composed of Ca(OH)_2_ generated from the hydrolysis of calcium carbide to produce acetylene, is an industrial waste. China was accounted for over 90% of the global output of calcium carbide, with the country being the world’s leading producer. Bai et al. [11] found that the high calcium content and alkalinity in CS had an activating effect of “calcium supplementation and alkalinity enhancement” on mineral admixtures. The pH value of a CS-saturated solution reached 12.7, and based on existing research, this paper also selected CS for the activation of silicoaluminate materials [12].

The construction-convenient PFSS is a low-energy engineering backfill material that can replace traditional compacted backfilling methods and solve the current accumulation of construction waste soils. However, the traditional PFSS preparation method mainly uses cement for solidification, which has the problem of high cost and carbon emissions. Alkali-activated cementitious material (AAM), an innovative and eco-friendly construction material, demonstrates significant advantages over traditional ordinary Portland cement (OPC) across various scientific aspects. These advantages are particularly evident in terms of durability [13], environmental benignity [14,15], mechanical performance [16], and energy-efficient production [17], positioning AAM as a promising candidate to potentially replace the OPC system in the future [18,19,20,21]. Therefore, substituting industrial waste for a portion of cement creates a cement-alkali-activated composite system, which is increasingly recognized as a mainstream approach for reducing cement usage [22,23].

Jawad et al. [24] found that using industrial waste to replace part of the cement can produce additives with early and late strengths that exceed those of cement. Wang et al. [25] used various industrial wastes to solidify soil based on the response surface method, and the resulting solidified soil had a 7 d curing strength higher than that of OPC-solidified soil. Li et al. [26] showed that replacing part of the cement with CS for solidifying clay can increase its strength to more than twice that of soil solidified with cement alone. Arulrajah et al. [27] demonstrated that industrial waste, represented by CS and FA, can replace cement to prepare a cost-effective and high-performance solidification material.

Based on the above research results, this paper conducted a performance study of CFGC-PFSS under the influence of different factors. At the same time, the microstructure and hydration products of PFSS were characterized by means such as X-ray diffraction (XRD), Fourier-transform infrared spectroscopy (FT-IR), thermogravimetric analysis (TGA), scanning electron microscopy (SEM), and energy dispersive spectrometry (EDS). The aim was to provide some theoretical guidance for the synergistic preparation of PFSS with industrial waste.

## 2. Raw Materials and Experimental Methods

### 2.1. Raw Materials

**Figure 1:**
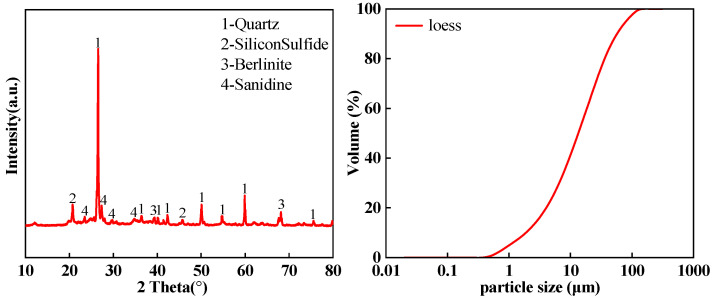
XRD diagram of soil sample and particle size analysis curve of soil sample.

**Figure 2:**
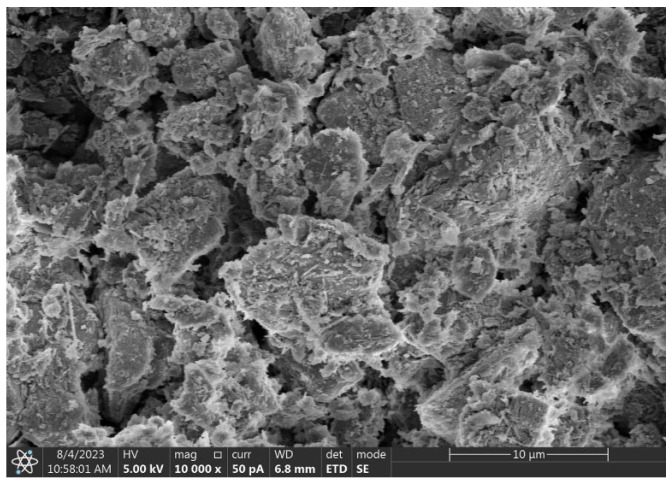
SEM image of loess (mag. ×10,000).

The cement used was P.O 42.5 ordinary Portland cement (brand name Lianshi), produced by Fujian Hongchang Concrete Co., Ltd. (Fuzhou, China). The GGBFS was S95-grade ground granulated blast furnace slag, produced by Henan Wuhu Technology Co., Ltd. (Wuhu, China); FA met the technical requirements of Class F Grade II, produced by Fujian Huadian Kemen Power Generation Co., Ltd. (Fuzhou, China); the loess, after crushing, was used after drying to a constant weight with particle sizes less than 2.36 mm, collected from the engineering waste soil of Fuzhou High-Tech Zone; the CS had a pH of 12.7, produced by Henan Wuhu Technology Co., Ltd. (Wuhu, China). The chemical composition of the loess, cement, GGBFS, FA, and CS are shown in Table 1, and the physical parameters of the soil sample is shown in Table 2. The XRD and particle size distribution curves of the soil samples are shown in Figure 1, and the SEM images are shown in Figure 2.

### 2.2. Experimental Testing Methods

#### 2.2.1. Specimen Molding and Curing

In accordance with the “Technical standard for placement of pre-mixed fluidized solidifying soil” (T/CECS 1073-2022) [28], pour the well-mixed PFSS into a 40 mm × 40 mm × 160 mm triple plastic mold, allowing it to consolidate naturally. Use a spatula to level the surface of the mold and cover it with plastic wrap. Place it under room-temperature conditions for curing between 24 and 36 h. After demolding, place it in a standard curing box (temperature (20 ± 2) °C, relative humidity above 95%) for curing until the required age for testing is reached before use.

#### 2.2.2. Liquidity Measurement

In accordance with the “Test methods for air-entrained mortars and grouts”, a hollow acrylic glass cylinder with an inner diameter of 80 mm and a height of 80 mm is used to determine the fluidity of the CFGC-PFSS.

#### 2.2.3. Mechanical Strength Determination

In accordance with the “ISO method for testing the strength of cement mortar” (GB/T 17671-2020) [29], a CDT 1305-2 microcomputer-controlled electronic universal testing machine is used to conduct flexural and compressive strength tests on the specimens at a rate of 50 N/S ± 10 N/S and 2400 N/S ± 200 N/S.

#### 2.2.4. SEM-EDS Analysis

The morphology of the CFGC-PFSS specimens at 7 d and 28 d was analyzed by an electron microscope of model Verios G4 UC (Thermo Fisher Scientific, Waltham, MA, USA). Additionally, after the 28 d compressive strength test, a sample of approximately 10 mm × 10 mm was taken from the PFSS specimen, sputter-coated with gold, and then the sample was transferred to the scanning electron microscope for testing. The energy-dispersive spectrometer is used to test the elemental content in the hydration products.

#### 2.2.5. XRD Analysis

The mineral composition of the CFGC-PFSS mortar specimens was analyzed by an X-ray diffractometer. of model Xpert 3 (Bruker, Billerica, MA, USA). The specimens were ground in a mortar and pestle until the sample fineness was below 0.075 mm, then the powder was dried and placed into the depression of a glass slide to form a 10 mm × 10 mm thin-film specimen. The test was conducted under conditions of 2θ ranging from 10° to 80°, with a step size of 0.02 and a scanning speed of 2°/min.

#### 2.2.6. FT-IR Analysis

The Fourier transform infrared spectrometer of model Nicolet IS10 (Thermo Fisher Scientific, Waltham, MA, USA) was used for FT-IR analysis, collected the PFSS specimens that had undergone mechanical strength testing and incorporated cementitious materials. After breaking them into pieces, we placed them in anhydrous ethanol to halt the hydration reaction of the cementitious materials and then placed them in an oven at 60 °C for 24 h. After the specimens were completely dried, we ground them into a powder and placed them into the Fourier transform infrared spectrometer for testing.

#### 2.2.7. TG Analysis

The STA 449F5 instrument (NETZSCH, Selb, Germany) is used for the TG analysis of CFGC-PFSS powder. Under a nitrogen atmosphere, we heated it at a rate of 20 °C/min from room temperature, 30 °C to 1000 °C, observed the change in the mass of the tested sample after heating, and recorded the curve drawn by the program when the mass changed.

### 2.3. Test Mix Proportion Design

In accordance with the “Technical standard for placement of pre-mixed fluidized solidifying soil” (T/CECS 1037-2022) [28], the strength requirement for PFSS at 28 d was between 0.4 MPa and 5.0 MPa, and the curing agent dosage was between 7% and 25%. In this study, the curing agent dosage was taken as 15% to 25% of the soil sample mass, and the water–solid ratio was between 0.46 and 0.54 for research. The mixing proportion of PFSS with the curing agent dosage was 15% and the water–solid ratio was 0.50 (shown in Table 3).

## 3. Results and Analysis

### 3.1. Determination of Water–Solid Ratio

**Figure 3:**
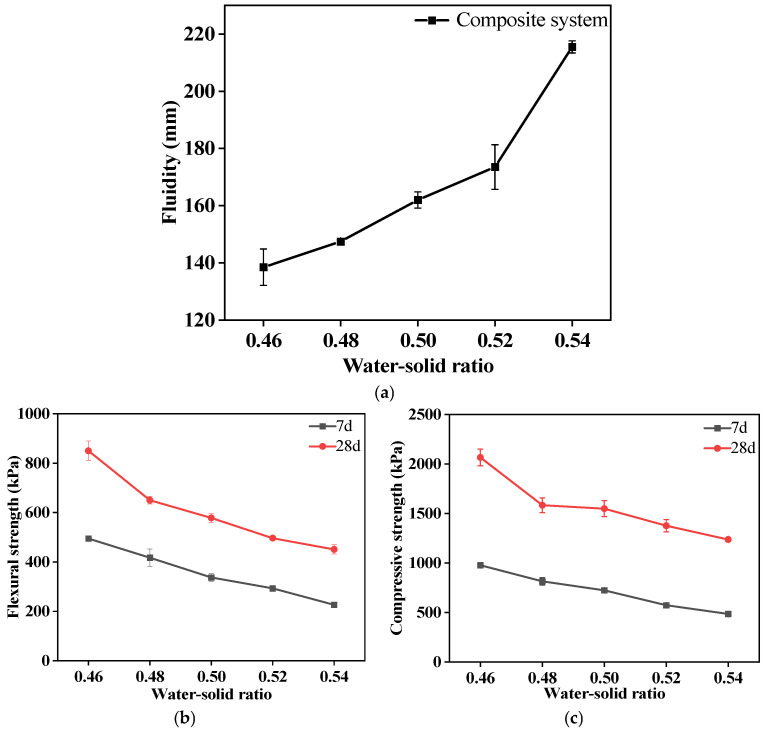
Effects of different water–solid ratios on the workability and mechanical strength of CFGC-PFSS: (**a**) the fluidity of PFSS under different water-solid ratios; (**b**) the flexural strength of PFSS under different water-solid ratios; (**c**) the compressive strength of PFSS under different water-solid ratios.

The fluidity of the PFSS mixture increases with an increase in the water–solid ratio (see Figure 3a). And as the water–solid ratio increased from 0.46 to 0.54, the fluidity of the PFSS mixture increased by 77 mm.

From Figure 3b,c, it can be observed that as the water–solid ratio increases from 0.46 to 0.54, the mechanical strength of the PFSS gradually decreases. The 7 d flexural strength decreased from 494.7 kPa to 226.0 kPa, and the 28 d flexural strength decreased from 850 kPa to 450.8 kPa. The 7 d compressive strength of the PFSS decreased from 977.1 kPa to 484.7 kPa, and the 28 d compressive strength decreased from 2066.0 kPa to 1236.5 kPa (all meet the minimum strength requirement of 0.4 MPa at the age of 28 d).

The selection of water is key to controlling the workability of PFSS. The dispersing action of water can disrupt the binding effect and friction between soil particles. Then, it will form a water film between them. The water film acts as a lubricant. And the thicker the film, the more it benefits the improvement of the workability of the mixture.

### 3.2. Determination of Curing Agent Dosage

**Figure 4:**
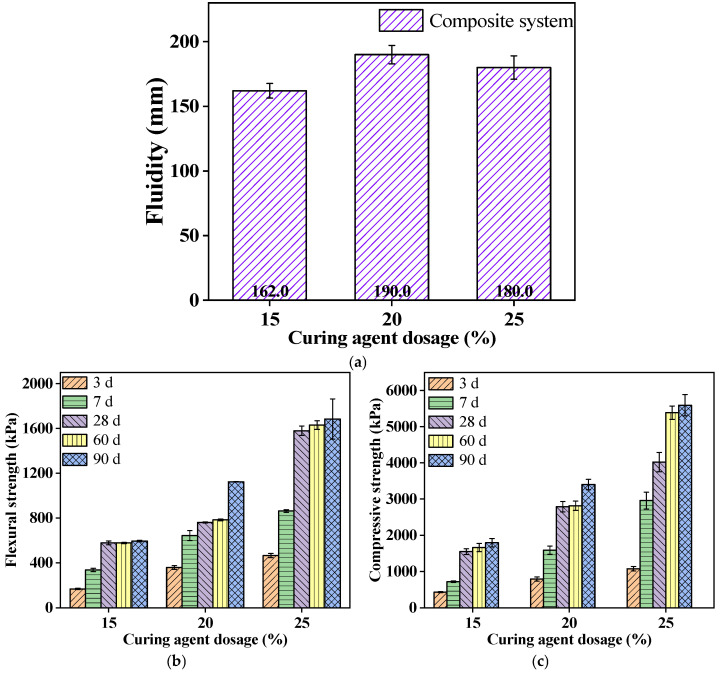
The influence of the curing agent dosage in CFGC-PFSS on fluidity and mechanical strength: (**a**) the fluidity of PFSS under different curing agent dosages; (**b**) the flexural strength of PFSS under different curing agent dosages; (**c**) the compressive strength of PFSS under different curing agent dosages.

The fluidity of the PFSS mixture decreases with an increase in the curing agent dosage (see Figure 4a); as the curing agent dosage increases from 15% to 25%, the fluidity increases by 18 mm.

From Figure 4b,c, it can be seen that as the curing agent dosage ranges from 15% to 25%, the mechanical strength of the PFSS gradually increases. Specifically, the 7 d flexural strength increased by 90.9% and 1.6 times; the compressive strength increased by 1.2 times and 3.1 times. The 28 d flexural strength increased by 31.7% and 1.7 times; the compressive strength increased by 80.1% and 1.6 times.

The increase in the curing agent dosage enhances the mechanical strength of PFSS, but it also increases the demand for water. When the water–solid ratio is constant, the increase in the curing agent dosage leads to an increase in the amount of water absorbed on the surface of the cementitious materials, which reduces the thickness of the water film and decreases the workability of the mixture. Among this, the adsorbed water reacts with the cementitious materials through hydrolysis, generating a large amount of the gel phase that encapsulates the soil particles, forming a stable “skeleton” and enhancing the mechanical strength of the PFSS.

### 3.3. Microscopic Analysis

#### 3.3.1. XRD Analysis

**Figure 5:**
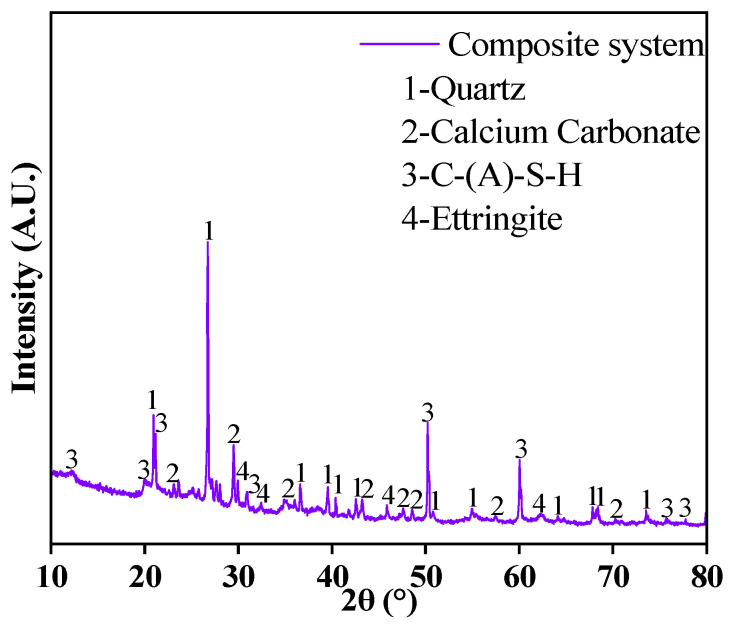
XRD analysis of composite system cementitious materials.

The phase analysis results of the hydration products of the composite cementitious material at 28 d are shown in Figure 5. As can be seen from the figure, there are four mineral phases present in the sample: quartz [SiO_2_], calcium carbonate [CaCO_3_], calcium aluminate silicate hydrate [C-A-S-H], and ettringite [AFt] diffraction peaks. Among them, SiO_2_ originates from the mineral phase of the raw materials and its presence may be due to partially unreacted cementitious materials. CaCO_3_ may be formed due to the carbonation reaction of Ca(OH)_2_ with CO_2_ in the air. The needle-like AFt and C-A-S-H gel are the main hydration products produced by the hydrolysis reaction of the composite cementitious materials, and the formation of these binding substances is the main source of the strength of the samples.

#### 3.3.2. FT-IR Analysis

**Figure 6:**
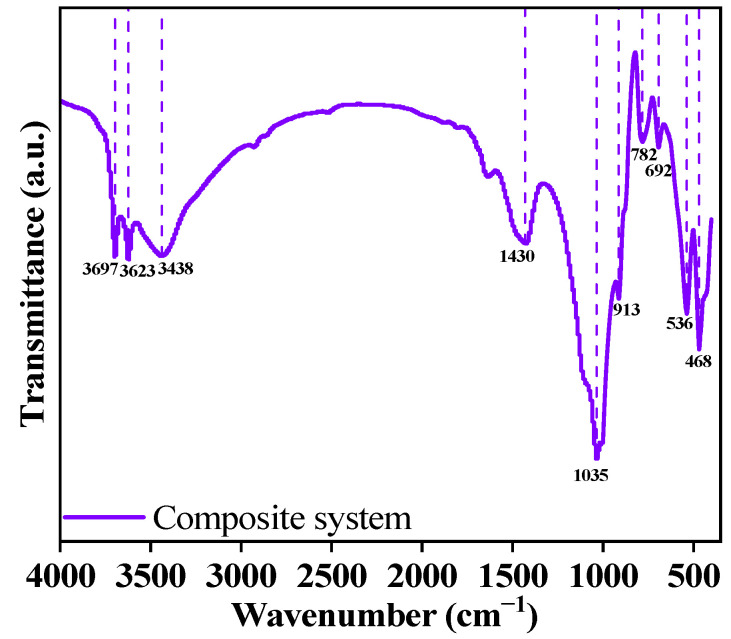
FT-IR analysis of CFGC-PFSS.

The FT-IR spectrum of the sample at 28 d is shown in Figure 6, clearly identifying 10 different peaks. The peaks near 3697 cm^−1^, 3697 cm^−1^, and 3438 cm^−1^ are typically associated with the stretching vibrations of hydroxyl groups (-OH) in the material [30]. The absorption peaks near 1430 cm^−1^ and 1035 cm^−1^ are attributed to the stretching vibrations of C-O bonds and the out-of-plane bending vibrations of CO_3_^2−^ [31,32], which originate from CaCO_3_ and HT, respectively. The absorption peak near 913 cm^−1^ is attributed to the stretching vibrations of Si(Al)-O bonds [31], while the absorption peaks near 782 cm^−1^ and 692 cm^−1^ are attributed to the Al-OH vibration peaks and the symmetric bending vibrations of Si-O-Si bonds in C-S-H, respectively [33]. The absorption peaks around 536 cm^−1^ and 468 cm^−1^ are caused by the out-of-plane and in-plane bending vibrations of Si(Al)-O bonds, mainly originating from the C-A-S-H gel [34].

#### 3.3.3. TG Analysis

**Figure 7:**
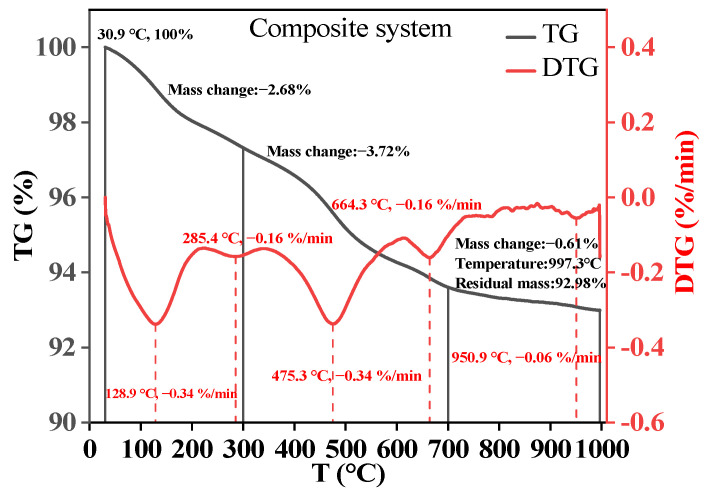
TG-DTG analysis of CFGC-PFSS.

The TG-DTG curve of the composite cementitious material after 28 d of curing is shown in Figure 7. It can be observed from the figure that there is a significant weight loss in the sample within the temperature range of 100–150 °C, which is mainly caused by the evaporation of free water within the cementitious paste [35]. The weight loss peak near 300 °C is primarily due to the decomposition of the C-A-S-H gel [35]. The weight loss between 450 and 500 °C is caused by the decomposition of Ca(OH)_2_ [36], and the weight loss of the paste within the range of 650–700 °C is due to the decomposition of calcite, indicating the presence of CaCO_3_ in the cementitious paste [37]. These results are consistent with the test results of XRD and FT-IR.

#### 3.3.4. Elemental Composition Analysis and Microscopic Morphology

**Figure 8:**
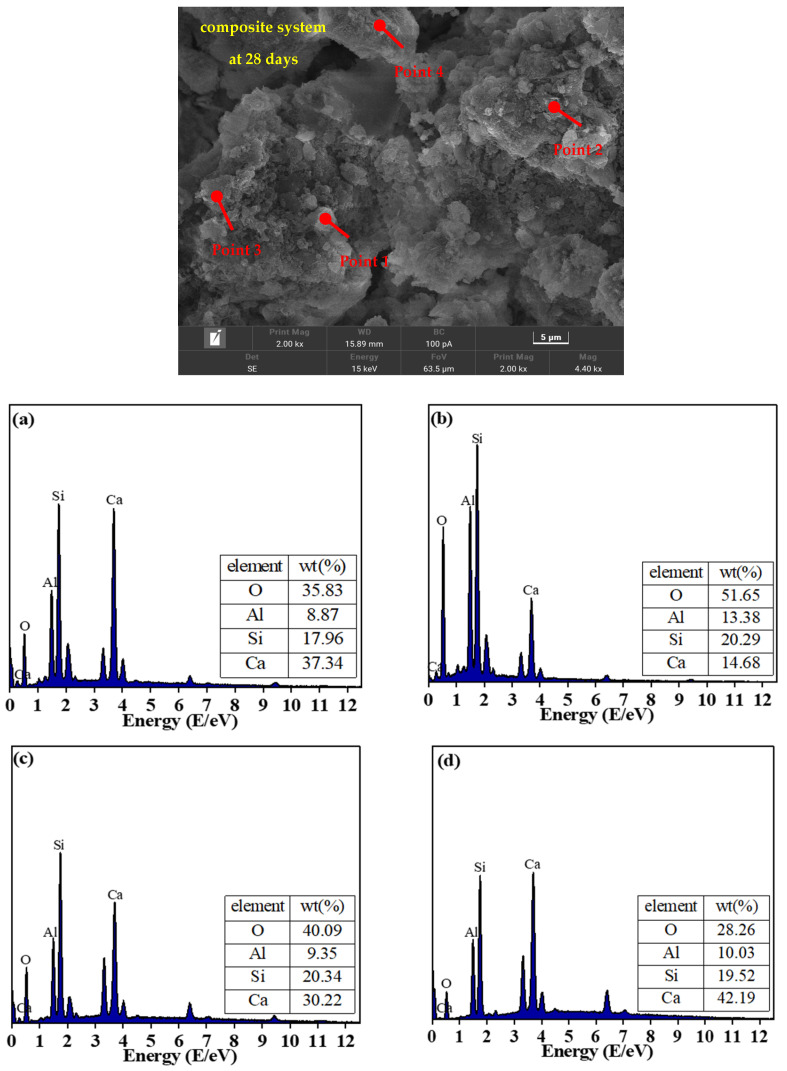
EDS point scanning analysis of CFGC-PFSS: (**a**) the EDS results at point 1; (**b**) the EDS results at point 2; (**c**) the EDS results at point 3; (**d**) the EDS results at point 4.

The EDS results of the CFGC-PFSS at 28 d are shown in Figure 8. It can be observed from the figure that the main elements in the 28 d sample include Ca, Si, Al, and O. Combined with the XRD results, it can be determined that the hydration products are C-S-H/C-A-S-H gels and needle-like AFt [38,39].

In conjunction with the EDS results, it can also be seen from the figure that in the early stages, a higher amount of Ca(OH)_2_ produces more needle-like C-S-H gels. However, as hydration proceeds and consumes Ca(OH)_2_, the needle-like C-S-H gels evolve into amorphous C-S-H gel. When the amorphous C-S-H gel has a low Ca/Si ratio, silicon dioxide in the C-S-H gel is replaced by aluminum, thereby forming the C-A-S-H gel [40].

**Figure 9:**
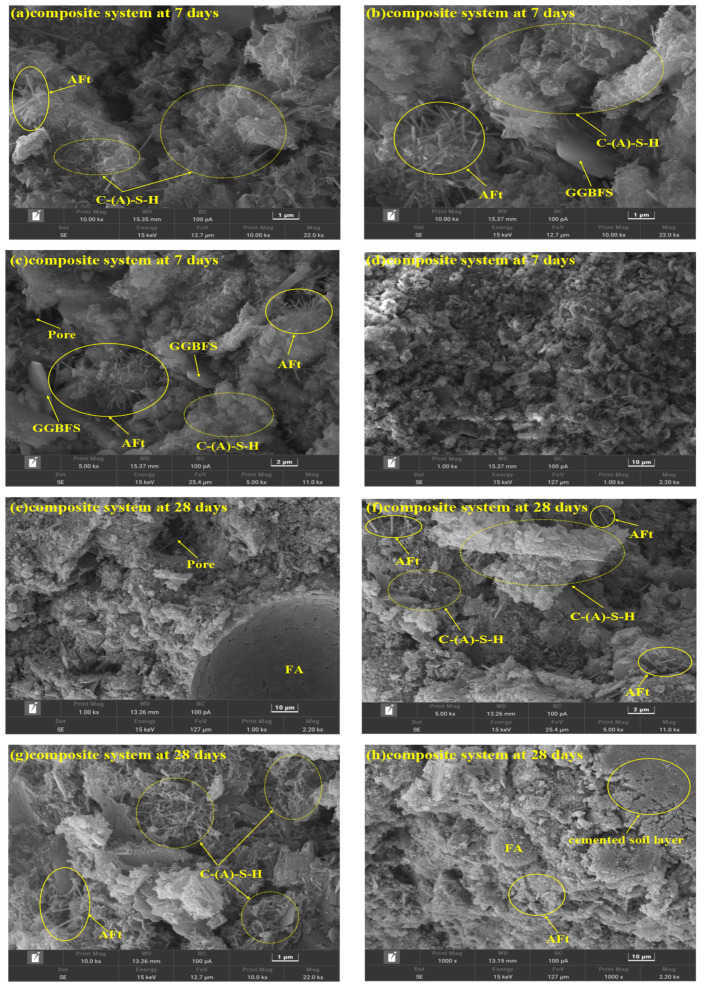
SEM analysis of CFGC-PFSS: (**a**–**d**) composite system at 7 days; (**e**–**h**) composite system at 28 days.

Figure 9 displays the SEM images of the CFGC-PFSS at 7 d and 28 d. It can be observed from the images that in the early stages of hydration, the gel has not yet developed into a dense structure, and there are a large number of cracks and pores present. As the curing age increases, the pores in the samples decrease, and the microstructure becomes more compact. Concurrently, as hydration progresses, the quantity of hydration products in the samples increase, and a large number of needle-like AFt and reticular C-S-H and C-A-S-H gels appear, which is consistent with the results shown by the FT-IR, TG-DTG, and EDS curves. The three cementitious materials coagulate to form a dense spatial reticular structure, encapsulating soil particles and forming a consolidated body with certain mechanical strength. Therefore, the mechanical strength of the samples significantly increase after 28 d of standard curing compared to 7 d.

## 4. Conclusions

In the current research, a cement-based composite curing agent was developed for soil solidification through the synergistic effect of various solid wastes. The workability and mechanical strength of the PFSS after solidification was studied, and the microstructure and hydration products at 28 d were characterized. The following conclusions were drawn:

(a) An increase in the water–solid ratio thickens the water film between soil particles. While a thicker water film enhances the fluidity of the mixture, it can compromise the bonding and friction between soil particles, leading to a decrease in mechanical strength.
(b) The addition of more curing agents leads to the formation of a substantial amount of the gel phase, which strengthens the mechanical properties of PFSS. However, the absorption of water by the surfaces of the large quantities of cementitious materials can reduce the thickness of the water film, thereby diminishing the fluidity of the mixture.
(c) Various micro-scale techniques indicate that under the influence of CFGC, a large amount of needle-like AFt, C-S-H gel, and C-(A)-S-H gel are formed to bind soil particles, achieving soil solidification. The formation of a large amount of hydrated gel is also the mechanism of strength formation in PFSS.
(d) The current CFGC curing agent had certain performances and benefits in soil solidification. Further tests on its durability and shrinkage properties will be conducted, and it will be further optimized.

## Figures and Tables

**Table 1:** Chemical composition of loess, cement, GGBFS, FA, and CS.

Raw Materials	SiO_2_	Al_2_O_3_	Fe_2_O_3_	K_2_O	TiO_2_	MgO	CaO	Na_2_O	Loss on Ignition
Loess	64.00	24.77	4.82	3.77	0.68	0.58	0.57	0.40	0.41
Cement	21.28	5.99	3.31	0.13	1.24	2.16	59.30	0.49	6.1
GGBFS	25.31	15.21	3.57	0.26	0.70	8.68	41.87	0.55	3.85
FA	47.65	34.63	6.71	1.40	1.47	0.56	5.48	0.29	1.81
CS	1.57	1.98	0.47	0.04	0.09	0.00	68.97	0.30	26.58

**Table 2:** Basic physical property indicators of soil samples.

Moisture Content/%	Density g/cm^3^	Liquid Limit/%	Plastic Limit/%	Plasticity Index	Liquidity Index	Cohesion/kPa	Internal Friction Angle/°
29.5	2.00	30.6	17.5	13.1	0.72	6.30	7.23

Note: IP = 13.1 satisfies 10 < IP ≤ 17, IL = 0.72 satisfies 0.25 ≤ IL ≤ 0.75, and the soil sample is silty clay and plastic soil.

**Table 3:** Design of mixing proportion for PFSS/g.

Cement/g	GGBFS/g	CS/g	FA/g	Loess/g	Water/g	Curing Agent Dosage/g
117	23	1.65	10	1000	575	150

## Data Availability

The original contributions presented in the study are included in the article, and further inquiries can be directed to the corresponding author.
